# Deficiency of B vitamins in women of childbearing age, pregnant, and lactating women in Brazil: a systematic review

**DOI:** 10.1186/s13643-025-02861-9

**Published:** 2025-05-16

**Authors:** Tatiane Salgado Galvão de Macedo, Michel Carlos Mocellin, Simone Augusta Ribas, Michelle Teixeira Teixeira, Alessandra da Silva Pereira, Gabriel Montalvão Palermo, Cintia Chaves Curioni

**Affiliations:** 1 Postgraduate Program in Food, Nutrition and Health, Institute of Nutrition, State University of Rio de Janeiro, Rio de Janeiro, RJ Brazil; 2 Department of Fundamental Nutrition, Nutrition School, Federal University of the State Do Rio de Janeiro, Rio de Janeiro, RJ Brazil; 3 Department of Public Health Nutrition, Nutrition School, Federal University of the State Do Rio de Janeiro, Rio de Janeiro, RJ Brazil; 4 Nutrition School, Federal University of the State of Rio de Janeiro, Rio de Janeiro, RJ Brazil; 5 Department of Social Nutrition, Institute of Nutrition, State University of Rio de Janeiro, Rio de Janeiro, RJ Brazil

**Keywords:** Women, Vitamin B complex, Vitamin B deficiency, Deficiency diseases, Brazil

## Abstract

**Objective:**

This study aimed to evaluate the prevalence of B-complex vitamin deficiencies in Brazilian women of childbearing age, pregnant women, and lactating women.

**Methods:**

This systematic review analyzed cross-sectional and cohort studies published up to August 2023 and indexed in MEDLINE, SciELO, LILACS, Scopus, Embase, Web of Science, and the Brazilian Digital Library of Theses and Dissertations. Additional data were obtained by contacting researchers from Brazilian public universities. Studies assessing deficiency rates using biochemical markers were included. Two reviewers independently selected studies, extracted data, and assessed methodological quality using the Joanna Briggs Institute tool.

**Results:**

Of the 3772 records identified, 13 studies were included. Only folate (*n* = 13), B12 (*n* = 11), and B6 (*n* = 1) deficiencies were investigated, and all studies were cross-sectional. B12 deficiency prevalence varied widely, reaching up to 29.4%. Folate deficiencies were generally low, with only one study reporting a rate as high as 37%. Other B-complex vitamins were insufficiently studied. Most studies had methodological limitations, particularly small sample sizes. The significant heterogeneity across studies limited the feasibility of a pooled quantitative meta-analysis.

**Conclusion:**

There is a clear need for more robust studies across all Brazilian regions to improve understanding of vitamin deficiency rates and to support effective nutritional interventions.

**Systematic review registration:**

PROSPERO CRD42020188474

**Supplementary Information:**

The online version contains supplementary material available at 10.1186/s13643-025-02861-9.

## Introduction

The World Health Organization (WHO) estimates that nearly two billion people worldwide have some form of micronutrient deficiency, primarily affecting women of childbearing age, pregnant women, and children in low- and middle-income countries [1–3]. Possible causes include poor diet quality, characterized by low quantities of vitamins and minerals contrasted with high consumption of ultra-processed foods [4], and infections and/or chronic diseases that may compromise food intake and nutrient absorption [5].

Among micronutrients, B-complex vitamins, particularly folic acid, vitamin B12, and vitamin B6, play a vital role in female reproductive health and fertility. Notably, these deficiencies often occur concurrently due to their complex interrelationships within metabolic pathways. A deficiency in one B-complex vitamin can impair the absorption and/or metabolism of others, which, in turn, is associated with an increased risk of adverse events for women and children, such as pregnancy-related anemia, maternal mortality, miscarriage, premature birth, and/or babies with low birth weight, neural tube defects, and cognitive problems [2, 6–8]. Additionally, these deficiencies have been associated with an increased risk of infertility due to hormonal changes that can contribute to the absence of ovulation and irregularities in the menstrual cycle [9–12]. The continuous use of oral contraceptives can also trigger deficiencies in micronutrients, such as folic acid and vitamins B2, B6, B12, C, and E [13, 14].

Studies also suggest that women in low- and middle-income countries (LMICs), including Brazil, particularly those of childbearing age and pregnant women, may be more susceptible to vitamin B deficiencies [2, 6, 15]. As a representative LMIC, Brazil faces challenges such as socioeconomic disparities, limited access to diverse and nutritious diets, and barriers to healthcare access, which may further exacerbate these deficiencies [16–18]. Therefore, it is essential to outline the prevalence of these deficiencies in this population. This assessment is crucial for supporting discussions on effective policies and strategies for monitoring, preventing, and treating these nutritional deficiencies adapted to the local context.

While some studies have investigated B-complex vitamin deficiencies in LMICs, comprehensive syntheses of these findings remain limited. There is a notable lack of systematic reviews focused on the Brazilian context, which limits the understanding of the specific factors influencing these deficiencies in local populations. This study aims to fill this gap.

In this context, the present study aims to identify, review, and synthesize findings from research investigating the prevalence of B-complex vitamin deficiencies among women of childbearing age, pregnant women, and lactating women in Brazil. Additionally, it seeks to explore potential factors associated with these deficiencies. Specifically, this systematic review was conducted to answer the following research question: “What is the prevalence of B-complex vitamin deficiencies in women of childbearing age, pregnant women, and lactating women in Brazil?”.

## Methods

This systematic review followed the recommendations of the Joanna Briggs Institute Manual of Evidence Synthesis (JBI) [19] and the checklist “Preferred reporting items for systematic reviews and meta-analyses — PRISMA” [20]. The protocol was registered in the PROSPERO database of systematic review registrations (CRD42020188474) [21]. The checklist PRISMA is presented in Additional File 1.

### Search strategy

We searched PubMed, SciELO, Lilacs, Scopus, Embase, Web of Science, and the Brazilian Digital Library of Theses and Dissertations databases to identify eligible studies published up to August 2023 using specific search strategies created for this purpose (available at Additional File 2). Since this study focused on the Brazilian population, it was crucial to search for other sources, such as manually checking the reference lists of selected articles and contacting researchers via email in the nutrition, medicine, and public health fields from all public universities in Brazil.

### Eligibility criteria

We included cross-sectional or cohort (baseline) studies published as original scientific articles, theses, or dissertations conducted in Brazil in any setting (primary care, hospitals, outpatient clinics, etc.). The study population comprised women of childbearing age (the period between menarche and menopause or women aged between 10 and 49 years), pregnant women, or lactating women. The studies had to present data on the prevalence of B-complex vitamin deficiency diagnosed by biochemical markers. There were no restrictions on date or language.

The exclusion criteria consisted of studies with samples containing a specific subset of the general population, such as patients with diseases or any pathological condition (except obesity associated with its comorbidities); individuals with conditions affecting digestion, absorption, metabolism, and daily vitamin requirements, such as postbariatric surgery and athletes; women with genetic polymorphisms affecting the body status of these vitamins; and studies in which the original authors were contacted for additional information to confirm eligibility but did not provide it.

### Study selection

We exported the results to the Rayyan QCRI web platform for reference management [22]. Duplicates were removed. Two reviewers (M. C. M. and T. S. G. M.) independently conducted the initial analysis of titles and abstracts. Those identified as potentially relevant received further scrutiny through full-text examination. In cases of disagreement, consensus was reached through discussion. We performed a pilot selection to assess the agreement between the two reviewers (M. C. M. and T. S. G. M.). The results demonstrated a 99% level of agreement, calculated through the kappa agreement index, which was 0.95 (standard error of 0.10).

### Data extraction

Two independent reviewers extracted data (M. C. M. and T. S. G. M.) using a Microsoft Office Excel spreadsheet created for this purpose. The following information was recorded: study identification; year and type of publication; location and period; study design; investigated study population and its characteristics (age, setting, proportion of obesity, malnutrition, chronic diseases, use of medications and vitamin supplements); sampling process; number of participants recruited and analyzed; investigated B-vitamin deficiencies and their diagnostic method; prevalence data and their corresponding 95% confidence intervals (CIs); or the number of cases for prevalence calculation. Post-extraction, the data underwent cross-checking to identify discrepancies, which were resolved by reconsulting the original study, followed by discussion and consensus among the reviewers. Data not available in the publication were requested from the original authors.

Two independent reviewers extracted data (M. C. M. and T. S. G. M.) using a Microsoft Office Excel spreadsheet created for this purpose. The following information was recorded: study identification; year and type of publication; location and period; study design; investigated study population and its characteristics (age, setting, proportion of obesity, malnutrition, or chronic diseases, use of medications and vitamin supplements); sampling process; number of participants recruited and analyzed; investigated B-vitamin deficiencies and their diagnostic method; prevalence data and their corresponding 95% confidence intervals (CIs); or the number of cases for prevalence calculation. Post-extraction, the data underwent cross-checking to identify discrepancies, which were resolved by reconsulting the original study, followed by discussion and consensus among the reviewers. Data not available in the publication were requested from the original authors.

### Assessment of methodological quality

Two reviewers independently assessed the methodological quality of the studies (M. C. M. and T. S. G. M.) using the “Critical Appraisal Instrument for Studies Reporting Prevalence Data” tool from the Joanna Briggs Institute — JBI [23]. The tool, which includes nine items concerning the selection and sampling process, adequate description of the study population, data analysis, diagnostic methods of the clinical condition of interest, and statistical analyses, was used. For the assessment of sample size, based on the equation proposed by Daniel (1999) [24], Naing et al. (2006) suggested that for an expected prevalence proportion of vitamin deficiencies of 10%, with a precision of 5%, the minimum sample size would be 138 women [25].

We classified the adequacy of each domain as adequate, inadequate, unclear, or not applicable. Any disagreements between reviewers were resolved through discussion until a consensus was reached.

### Data synthesis and statistical analysis

A narrative description of the results was generated and is presented separately for pregnant women, lactating women, and women of childbearing age, as well as for each vitamin B evaluated. Despite the low quality of the studies and the methodological differences among the studies, we performed a meta-analysis following our original plans to summarize the rates and test variables that impute heterogeneity to the analysis.

The overall proportion of each vitamin B deficiency and the respective 95% confidence interval were calculated using the generic method of the inverse variance with a random effects model. The Freeman-Tukey (1950) double arcsine transformation was applied to stabilize variance. This statistical approach enables the inclusion of studies with no observed cases of B-complex vitamin deficiency (i.e., zero-event studies) in the meta-analysis. Heterogeneity was initially assessed by visual inspection of the forest-plot graphs and by Cochran’s Q test with a significance level of *α* = 0.1, considering the low power of this test. The *I*^2^ test was also used, which quantifies the inconsistency between studies to assess the impact of heterogeneity in the meta-analysis, considering *I*^2^ values between 25 and 50% are considered low, 50–75% moderate, and 75% or higher.

For each subpopulation and each B vitamin with at least two studies available, the analysis was stratified by the following factors: decade of data collection, age group of the population studied (adolescents and/or adults), Brazilian geographic region, cutoff points used to diagnose vitamin deficiency, and, in the case of folate, whether data were collected before or after the implementation of the Brazilian National Flour Fortification Program with folic acid (initiated in 2004).

Statistical analyses were performed using STATA vs. 16.0 (StataCorp LLC, TX, USA), using the Metapreg data package. For the calculation, we used the number of cases with the event and the total number of women investigated for deficiency in each interest group.

## Results

**Fig. 1:**
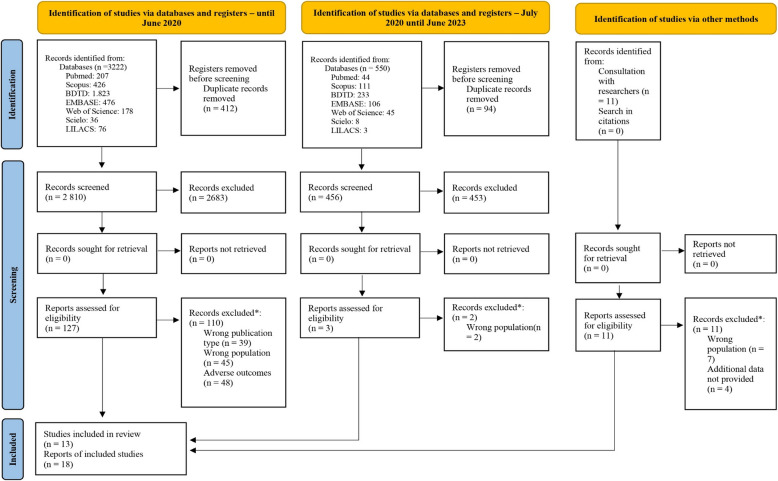
PRISMA flow diagram for the process of the search and study selection

Initially, we identified 3772 records in the databases and 11 through consultation with researchers. After removing duplicate publications among those found in the databases, 3266 remained. We completely evaluated 130 publications retrieved from the databases, along with 11 additional records identified through researchers. Following this analysis, 18 publications representing 13 unique studies were included in this review [26–38] (Fig. 1).

Three studies had multiple publications: (1) Cavalcanti et al. (thesis [28] and scientific article [39]) and the publications by Porto [40] and Dantas [41] used the same sample for their analyses and were considered as a single study, (2) the studies by Barnabé [27, 42], and (3) the studies by Steluti [36, 43] corresponded to two publication formats (thesis/dissertation and scientific article).

All included publications reported cross-sectional designs [26–38]. The Southeast Region of Brazil was the geographic region with the highest number of investigations [26, 27, 29, 31–33, 36], primarily conducted between the 2010 s and 2020 s [27, 28, 30, 32, 33, 35, 36, 38], with study samples sourced from primary health care facilities and hospitals [27, 29, 31, 34, 35, 39]. Folate data were available in all studies [26–38], 11 provided information on vitamin B12 [26–29, 31–34, 36–38], and only 1 study included data on vitamin B6 [36]. The sample size ranged from 23 to 1330 participants, totaling 4116 women across the 13 studies. Eight studies focused on women of childbearing age who were not pregnant or lactating [26, 28, 30, 32–34, 36, 37], four with pregnant women [27, 31, 34, 35], and three were carried out with lactating women [27, 29, 34] (Table 1).

**Table 1:** Characteristics and results of the included studies

Author, year/state	Sample source	Target population	Age (years)^a^		B vitamins assessed		Method for biomarker diagnosis		Number of participants analyzed (*n*)		Value adopted for deficiency diagnosis B12 (pg/mL) Folate (ng/mL) B6 (nmol/L)		Prevalence of deficiencies (95% *CI*)	
Barbosa et al., 2008 [26]/SP	Randomly selected volunteers	WCA	30.9 (29.3–32.6)^b^		B12		CLIA		102		< 200 < 244 < 350		10.0 (5.0–17.0) 20.6 (13.9–29.4) 52.9 (43.3–62.3)	
					SF						< 3 < 6		2.0 (0.0–7.0) 37.0 (28.0–47.0)	
					EF						< 160		2.0 (0.5–6.9)	
Barnabé, 2014 [27]/SP	Collection service at the Clinical Hospital of Unicamp	PW	14–43		B12		ECLIA		291		< 200		8.0 (5.0–12.0)	
					SF						< 4		0.0 (0.0–0.02)	
		LW	14–40		B12				54		< 200		2.0 (0.0–10.0)	
					SF						< 4		0.0 (0.0–7.0)	
Cavalcanti, 2018 [28]/PE	Outpatient Gynecology Clinic of the Municipal SUS Network	WCA	15–45		B12		ECLIA		1156		< 200		7.0 (6.0–9.0)	
					EF				1171		< 140		0.09 (0.02–0.48)	
Donangelo et al., 1989 [29]/RJ	Public hospital/maternity	LW	25.9 ± 5.6^c^		B12		Radioisotope dilution assay		LW ≤ 30 dpp: 35		< 150		29.0 (15.0–46.0)	
									LW > 30 dpp: 30				0.0 (0.0–12.0)	
					SF				LW ≤ 30 dpp: 36		< 3		11.0 (3.0–26.0)	
									LW > 30 dpp:31				26.0 (12.0–45.0)	
Oliveira et al., 2017 [30]/RS	Pelotas 1982 Birth Cohort	WCA	22–23		SF		Immunoassay		1330		< 3		0.0 (0.0–1.0)	
											< 4		3.0 (2.0–4.0)	
		OB							128		< 3		0.0 (0.0–3.0)	
											< 4		2.0 (0.0–7.0)	
Paiva et al., 2003 [31]/SP	Two public hospitals	PW	14–32		B12		Immunoassay		36		< 197		22 (10.0–39.0)	
					EF		Ion capture method		28		< 228		25.0 (12.7–43.4)	
					SF				33		(…)		(…)	
Palchetti et al., 2017 [32]/SP	General urban population of the city of São Paulo	WCA	≥ 20		B12		Microbiological method		62		< 200		0.0 (0.0–6.0)	
					SF						< 3.0		2.0 (0.0–9.0)	
Palchetti et al., 2022 [33]/SP	Subsample of ISA-Capital 2015	WCA	20–49		B12		ECLIA		99		< 200		29.4 (20.4–40.2)	
					SF						< 4		0.8 (0.1–5.5)	
Pinto et al., 1973 [34]/DF	Only hospital in the city of Sobradinho	WCA	(…)		B12		Microbiological method		30		< 140		0.0 (0.0–12.0)	
					SF						< 4		10.0 (2.0–27.0)	
		PW			B12				PW ≤ 20 WG: 33		< 140		0.0 (0.0–10.0)	
									PW > 20 WG: 46				9.0 (2.0–21.0)	
					SF				PW ≤ 20 WG: 28		< 4		12.0 (3.0–27.0)	
									PW > 20 WG: 45				24.0 (13.0–39.0)	
		LW			B12				35		< 140		6.0 (0.0–20.0)	
					SF						< 4		54.0 (37.0–71.0)	
Santos, 2016 [35]/AC	Primary health care prenatal services	PW	13–40		SF		Fluorimmunoassay		506		< 3		0.0 (0.0–1.0)	
Steluti, 2014 [36]/SP	Subsample of ISA-Capital 2008	WCA	12–49		B12		B12: CLIA SF; B6: HPLC		143		< 200		20.0 (15.0–26.0)	
					SF				205		< 3		1.0 (0.0–4.0)	
					B6				143		< 20		1.9 (0.6–6.2)	
			12–19		B12				69		< 200		13.0 (7.0–23.0)	
					SF						< 3		3.0 (0.0–10.0)	
					B6						< 20		0.0 (0.0–5.3)	
Tavares et al., 2004 [37]/PA	Parkatêjê Indigenous people	WCA	≥ 20		B12		CLIA		26		< 190		0.0 (0.0–13.0)	
					SF						< 3		15.0 (4.0–35.0)	
Tedesco et al., 2016 [38]/PR	Private bariatric surgery clinic	OB	18–49		B12		(…)		23		< 140 < 200		4.3 (0.8–21.0)4.3 (0.8–21.0)	
					SF						< 3 < 4		34.8 (18.8–55.1)65.2 (44.9–81.2)	

(…) missing information, *AC* acre, *B6* serum vitamin B6, *B12* serum vitamin B12, *CLIA* chemiluminescence immunoassay, *DF* Distrito Federal, *ECLIA* electrochemiluminescence immunoassay, *EF* erythrocyte folate, *HPLC* high-performance liquid chromatography, ISA-Capital ISA-Capital: Health Survey of São Paulo, *LW*, lactating women, *LW* > 30 dpp, lactating women > 30-day postpartum, *LW* ≤ 30 dpp, lactating women ≤ 30-day postpartum, *OB* women of childbearing age with obesity, *PA* Pará, *PE* Pernambuco, *PR* Paraná, *PW* pregnant women, *PW* > 20 WG, pregnant women > 20-week gestation, *PW* ≤ 20 WG, pregnant women ≤ 20-week gestation, *RJ* Rio de Janeiro, *RS* Rio Grande do Sul, *SF* serum folate, *SP* São Paulo, *SUS* Unified Health System, *WCA* women of childbearing age

^a^Age presented in range

^b^Geometric mean (95% *CI*: 95% confidence interval)

^c^Mean and standard deviation

### Vitamin B12

Among the studies that evaluated vitamin B12 deficiency, the highest prevalence observed in women of childbearing age was 29.4% (95% *CI*: 20.4–40.2), reported in a study using a representative subsample from a population-based survey [33], applying the most frequently used cutoff point for deficiency (< 200 pg/mL). In studies with population-based data, only two used representative samples. Both were conducted using data from the Health Survey of São Paulo (ISA-Capital SP) with women of childbearing age: Steluti et al. (2014) [43], who analyzed data from the 2008 edition, and Palchetti et al. (2022) [33], who examined data from 2014. The first study reported a prevalence of vitamin B12 deficiency of 20.0% (95% *CI*: 15.0–26.0), while the second found an even higher prevalence in the same population, increasing to 29.4% (95% *CI*: 20.4–40.2) in 2014.

In studies without representative samples, the highest prevalence was 10.0% (95% *CI*: 5.0–17.0), found in Barbosa et al. (2008) [26], which also used the same cutoff point. This study additionally tested stricter cutoffs and reported even higher prevalence rates as the threshold for deficiency increased. In two studies, the deficiency rate was null [34, 37].

Among pregnant women, the highest prevalence was observed in a study conducted in 2003 at two public hospitals in São Paulo, reaching 22.0% (95% *CI*: 10.0–39.0) [31]. Conversely, the study by Pinto et al. (1973) found no deficiency among pregnant women with gestational age ≤ 20 weeks [34].

Among lactating women, the highest prevalence was reported in a study conducted in 1989 at a public maternity hospital in the city of Rio de Janeiro, which found a prevalence of 29.0% (95% *CI*: 15.0–46.0) in lactating women ≤ 30 days postpartum [29] (Table 1).

**Fig. 2:**
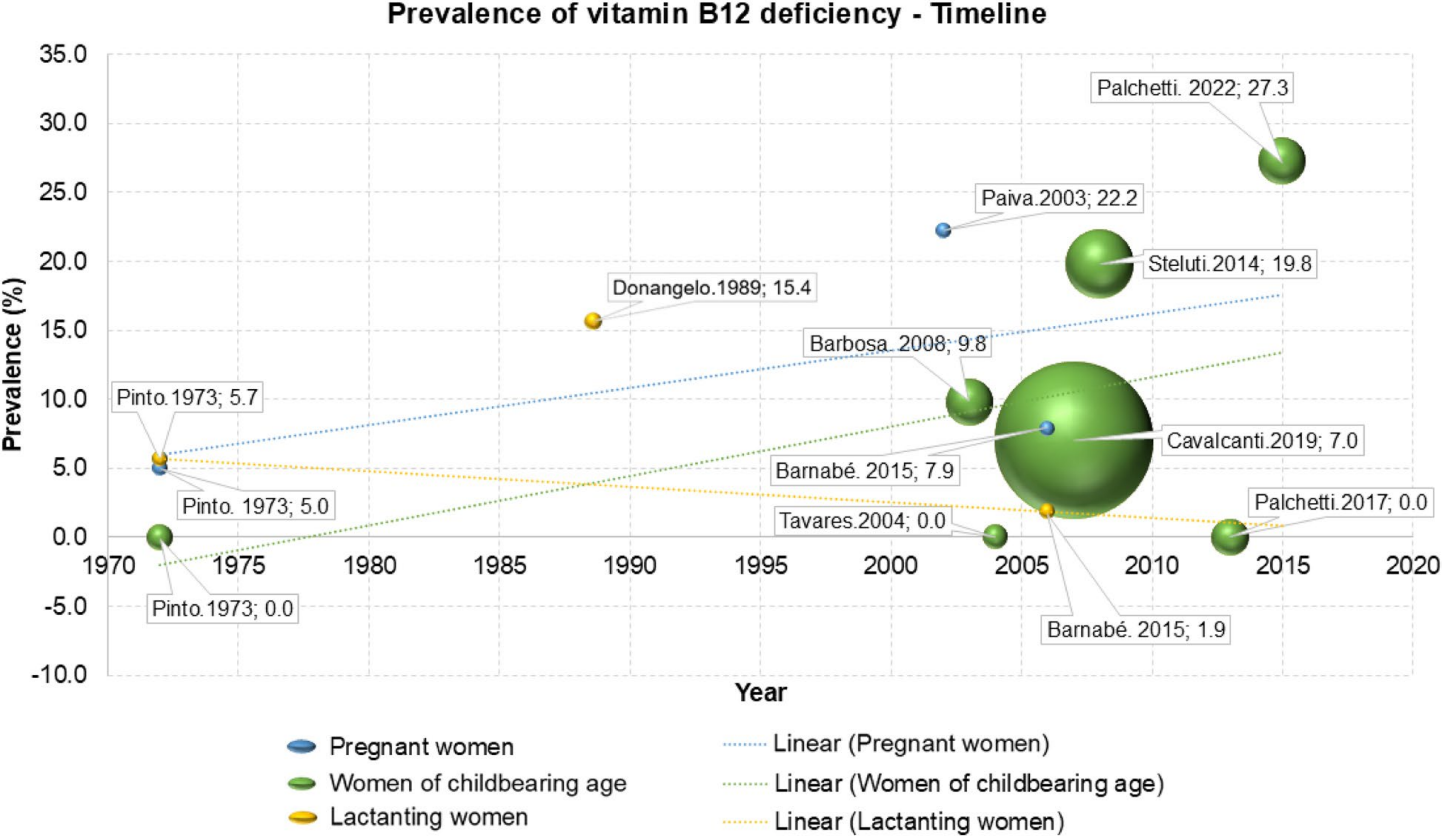
Prevalence of vitamin B12 deficiency — timeline

Analyzing the prevalence of vitamin B12 deficiency across studies according to the year of data collection, it appears that the prevalence of hypovitaminosis B12 has increased over time among pregnant women and women of childbearing age (Fig. 2).

### Folate

Among the studies with representative samples based on different editions of the ISA-Capital survey, both reported very low prevalence rates of serum folate deficiency in women of childbearing age: 1.0% (95% *CI*: 0.0–4.0) [43] and 0.8% (95% *CI*: 0.1–5.5) [33].

Considering the lack of standardization in the cutoff points adopted for diagnosing deficiency in the studies, Barbosa et al. (2008) [26] found the highest prevalence among women of childbearing age. This study, conducted with 102 randomly selected volunteers, reported a prevalence of 37.0% (95% *CI*: 28.0–47.0), using a cutoff point of < 6 ng/mL. It is worth noting that all other studies used a cutoff point for diagnosing serum folate deficiency of < 3 or < 4 ng/mL. One study [30] tested different cutoff points for defining serum folate deficiency. When using a threshold of < 3 ng/mL, no cases of deficiency were identified. However, when the cutoff was increased to < 4 ng/mL, the prevalence was 3.0% (95% *CI*: 2.0–4.0). For those with the most utilized cutoff point, the highest prevalence was 15.0% (95% *CI*: 4.0–35.0) in a study conducted in the Parkatêjê indigenous community [37].

Among pregnant women, the highest prevalence was 24.0% (95% *CI*: 13.0–39.0) in those with a gestational age greater than 20 weeks in a study conducted in the Distrito Federal in 1973 [33]. The highest rate of deficiency found in lactating women was 54.0% (95% *CI*: 37.0–71.0) in the same study [34] (Table 1).

Concerning erythrocyte folate, which was assessed in only three studies, the reported prevalence rates were 2.0% (95% *CI*: 0.5–6.9) [26] and 0.09% (95% *CI*: 0.05–0.48) [39] among women of childbearing age and 25.0% (95% *CI* 12.7–43.4) among pregnant women [31] (Table 1).

### Vitamin B6

The only study that evaluated vitamin B6 used a subsample from the ISA-Capital SP 2008 and reported a prevalence of vitamin B6 deficiency in adult women of childbearing age of 1.9% (95% *CI* 0.6–6.2) [36] (Table 1).

### Women of childbearing age with obesity

Two studies included women of childbearing age with obesity [30, 38]. One of these studies focused exclusively on women undergoing preoperative bariatric surgery [38], while the other included a broader sample of women of childbearing age, with results specifically stratified for those with obesity [30]. Only the study by Tedesco et al. [38] analyzed vitamin B12, revealing a prevalence of deficiency at 4.3% (95% *CI* 0.8–21.0), using cutoff points of < 140 pg/mL and < 200 pg/mL. Regarding the use of a serum folate concentration < 3 ng/mL, one study showed no deficiency [30], while the other reported a prevalence of 34.8% (95% *CI* 18.8–55.1) [38]. For < 4 ng/mL, the prevalence increased to 2.0% (95% *CI* 0.0–7.0) [30] and 65.2% (95% *CI* 44.9–81.2) [38], respectively (Table 1).

The results of the meta-analysis are presented in Additional File 3. It is important to emphasize that, rather than focusing solely on a single overall deficiency rate, identifying variables that may influence the global prevalence is more relevant. The subgroup analysis showed that none of the variables considered as potential effect modifiers significantly altered the overall results. However, these variables did contribute to a reduction in heterogeneity, indicating that they play a role in explaining the variability observed among studies.

### Methodological quality of the included studies

Table 2 presents the methodological quality of the included studies. None of the studies met all criteria classified as adequate. Notably, seven studies [27, 30, 32, 33, 36–38] lacked adequacy in the target population domain as they represented only a population subset. Only one study [28] adequately presented the sampling process, employing random selection. Most studies [26, 27, 29, 31–34, 37, 38] had sample sizes considered unsatisfactory. Likewise, among the studies with inadequate sample sizes, four out of nine [27, 29, 31, 34] reported losses of more than 5% of the recruited individuals, analyzed for the outcomes of interest, indicating significant selection and adherence biases. Only four studies provided detailed population and study location information [27, 28, 30, 35].

**Table 2:** Critical assessment of methodological quality of studies included in the systematic review

	Q1	Q2	Q3	Q4	Q5	Q6	Q7	Q8^a^	Q9
Barbosa, 2008 [26]	**✓**	✘	✘	✘	**✓**	**✓**	U	N/A	**✓**
Barnabé, 2014 [27]	✘	✘	✘	**✓**	**✓**	**✓**	**✓**	N/A	✘
Cavalcanti, 2018 [28]	**✓**	**✓**	**✓**	**✓**	**✓**	**✓**	✘	N/A	**✓**
Donangelo, 1989 [29]	**✓**	✘	✘	✘	**✓**	**✓**	✘	N/A	✘
Oliveira, 2017 [30]	✘	N/A	**✓**	**✓**	**✓**	**✓**	✘	N/A	**✓**
Paiva, 2003 [31]	**✓**	✘	✘	✘	**✓**	**✓**	✘	N/A	✘
Palchetti, 2017 [32]	✘	✘	✘	✘	**✓**	**✓**	**✓**	N/A	**✓**
Palchetti, 2022 [33]	✘	✘	✘	✘	**✓**	**✓**	**✓**	N/A	**✓**
Pinto, 1973 [34]	**✓**	✘	✘	✘	**✓**	**✓**	✘	N/A	✘
Santos, 2016 [35]	**✓**	U	**✓**	**✓**	**✓**	**✓**	**✓**	N/A	✘
Steluti, 2014 [36]	✘	✘	**✓**	✘	**✓**	**✓**	**✓**	N/A	**✓**
Tavares, 2004 [37]	✘	✘	✘	✘	**✓**	**✓**	U	N/A	**✓**
Tedesco, 2016 [38]	✘	N/A	✘	✘	**✓**	**✓**	U	N/A	**✓**
Total (%)	46.2	7.7	30.8	30.8	100	100	38.5	0	61.5

*Q1* — Was the sample frame appropriate to address the target population (women of childbearing age, pregnant women, or lactating women from a specific location/city/state/region of Brazil)? Q2 — Were study participants sampled in an appropriate way? Q3 — Was the sample size adequate? Q4 — Were the study subjects and the setting described in detail? Q5 –— Was the data analysis conducted with sufficient coverage of the identified sample? Q6 — Were valid methods used to identify the deficiency of the B complex vitamin studied? Q7 — Was the vitamin B complex deficiency studied and measured in a standard and reliable way for all participants? Q8 — Was there appropriate statistical analysis? Q9 — Was the response rate adequate, and, if not, was the low response rate managed appropriately?

✓ yes, ✘ no, *N/A* not applicable, *U* unclear

^a^All were rated as “NOT APPLICABLE,” as statistical data were not extracted; only the number of cases and individuals was evaluated. The prevalence was subsequently calculated

## Discussion

### Summary of key findings

This review systematically evaluated and analyzed the methodological quality of studies reporting the prevalence of B-complex vitamin deficiencies in women of childbearing age, pregnant women, and lactating women in the Brazilian population. The prevalence of vitamin B12 deficiency varied considerably across the included studies, ranging from 0 to 29.4% among the three groups of interest. The highest prevalence was reported among women of childbearing age in a study based on a population-representative sample [33]. Regarding serum folate deficiency, using the most adopted cutoff point of 3 ng/mL, prevalence estimates ranged from 0.0 to 26.0%, with the highest rate observed among lactating women. However, most studies reported lower prevalence rates for folate deficiency. Among women of childbearing age with obesity, one study reported notably high rates of folic acid deficiency: 34.8% when using a cutoff of < 3 ng/mL and 65.2% with a cutoff of < 4 ng/mL [38].

### Comparison to existing research

Notably, the highest prevalence of vitamin B12 deficiency (29.4%) was reported in a study that analyzed data from the 2014 ISA-Capital survey [33], based on a population-representative sample. In comparison, an earlier study using data from the 2008 edition of the same survey reported a lower prevalence of 20.0% [43]. These findings suggest a possible increase in vitamin B12 deficiency over time in this population, reinforcing the importance of robust and representative methodologies to generate accurate and generalizable estimates. This observed trend also underscores the urgent need for evidence-based public policies aimed at the prevention and control of this nutritional deficiency.

In Brazil, current food supplementation programs do not include vitamin B12, prompting a crucial need for policymakers to initiate discussions on the imperative of mandatory supplementation, particularly for pregnant women. Furthermore, contemplating the fortification of flour with vitamin B12 emerges as a preventive strategy for addressing B12 hypovitaminosis, a practice already implemented in certain countries in Latin America and North America [44]. These recommendations are particularly relevant given the increasing prevalence of vegetarians in the Brazilian population [45].

When compared to findings from other countries, the results observed in Brazil in women of childbearing age align with those reported in several Latin American nations, such as Colombia [46], Guatemala [47], Belize [48], and Mexico [49], as well as countries from other regions, including the UK [50], China [51], and Iran [52]. A study in China revealed a higher vitamin B12 deficiency, affecting 45.5% of the population among women of childbearing age [53]. Studies conducted in India among pregnant women revealed even higher rates, ranging from 46 to 73% [54–59]. Supplementation has proven effective in reducing vitamin B12 deficiency. A study conducted in New Zealand demonstrated that B12 supplementation decreased the prevalence from 48 to 30% in 6 months. Such evidence highlights the relevance of preventive supplementation strategies, especially among groups more susceptible to deficiency [60].

In contrast to vitamin B12, folate deficiency showed a lower prevalence across all three groups of interest. Although the erythrocyte folate concentration is considered a more reliable indicator of folate adequacy than serum folate concentration, it is notable that most included studies evaluated the serum folate concentration, reflecting recent changes in nutrient intake [61]. It is important to highlight that those studies not identifying serum folate deficiency in pregnant women often reported that about half of the participants used some form of vitamin supplement, including isolated folic acid [27, 35].

Internationally, folic acid supplementation is widely recommended to prevent neural tube defects, with the World Health Organization (WHO) and the Ministry of Health (MS) recommending folic acid supplementation at a daily dose of 400 µg for at least 30 days before conception and throughout the first trimester of pregnancy to prevent neural tube defects. Ideally, this supplementation should continue until the end of pregnancy to prevent megaloblastic anemia due to folate deficiency in the diet [62–64]. The widespread adoption of universal supplementation played a role in achieving adequate folate concentrations in almost all pregnant women. In Brazil, the enrichment of wheat and corn flour with iron and folic acid is governed by Resolution RDC No. 344/2002, a public policy implemented by mid-2004, to reduce the prevalence rates of iron and folate deficiencies [65]. The public policy appears to have contributed positively to reducing deficiency prevalence.

The results reported by one of the studies that evaluated women of childbearing age with obesity may not accurately reflect the general population, as the participants were undergoing preoperative preparation for bariatric surgery and may have been following energy-restricted diets, potentially leading to inadequate micronutrient intake [66–68]. Another study, conducted with a birth cohort sample, obtained much lower values: 0.0% and 2.0%, respectively [30]. Compared with eutrophic adults, adults with obesity have different micronutrient requirements, possibly greater [67–69]. Unfortunately, our findings could not contribute to this issue.

Despite these efforts, a study conducted among the Parkatêjê indigenous people showed a 15.0% prevalence of serum folate deficiency in women of childbearing age [37]. Research on this tribe has exposed a significant cultural shift over the past two decades, involving modifications in their nutritional patterns and a notable transition towards processed foods, leading to elevated rates of overweight and obesity, particularly among women [37, 70, 71]. New research should be conducted to investigate possible nutritional deficiencies and their consequences among indigenous people, considering the lack of attention given to this population in scientific publications.

Special attention should be given to the physiological changes during pregnancy, such as the hemodilution observed in the second trimester, which can affect serum concentrations of vitamin B12. This alteration may lead to an overestimation of deficiency rates if standard adult cutoff points are applied without appropriate adjustments. Notably, the cutoff values used in studies involving pregnant and lactating women were generally the same as those applied to women of childbearing age, despite the distinct physiological conditions of these groups [72].

A consensus on the appropriate cutoff points for identifying deficiencies in these nutrients is still lacking, and values may vary depending on the analytical method used to measure concentrations. For serum folate, experts commonly recommend a cutoff of 3 ng/mL [73–75], although some studies suggest that deficiency may begin at values below 4 ng/mL [76, 77]. For erythrocyte folate, concentrations between 140 and 150 ng/mL are generally considered indicative of deficiency [73, 75, 76, 78]. Serum concentration of vitamin B12 is widely used in epidemiological studies as a cost-effective and accessible biomarker to assess vitamin B12 status. Many studies suggest that values below 200 pg/mL indicate deficiency [73, 75, 76, 79–81]. However, despite the increased risk of vitamin B12 deficiency during pregnancy, the cutoff values applied are often the same as those used for the general adult population, which may not accurately reflect the needs and physiological variations in pregnant individuals [44, 76]. To minimize the risk of fetal malformations and ensure adequate maternal status, experts propose that ideal concentrations should exceed 400 ng/mL for erythrocyte folate [61], 7 ng/mL for serum folate, and 400 pg/mL for vitamin B12 [75].

### Strengths and limitations

Some limitations should be taken into account when interpreting the findings of this review. The heterogeneity in study methodologies, sampling strategies, data collection procedures, population characteristics, publication periods, and reported outcomes limited the feasibility of conducting a quantitative meta-analysis. Additionally, although the critical appraisal revealed methodological weaknesses in several studies—particularly concerning sampling strategies—the inclusion of diverse study designs allowed for a broader overview of the topic. The predominance of small samples from healthcare settings in specific municipalities may restrict the generalizability of some findings.

Despite these limitations, this review demonstrates several notable strengths. To the best of our knowledge, it represents the first comprehensive synthesis specifically focused on the prevalence of B-complex vitamin deficiencies among women of childbearing age, pregnant women, and lactating women in Brazil. The inclusion of studies that assessed vitamin deficiencies exclusively through biochemical markers enhanced the reliability and accuracy of the findings. Furthermore, the implementation of a rigorous and extensive search strategy, including a broad range of publication types beyond peer-reviewed articles, contributed to the overall comprehensiveness and strength of the evidence base evaluated in this review.

### Implications for policy and practice

Additionally, we observed either a complete absence or a very limited number of studies investigating the prevalence of deficiencies in specific B-complex vitamins, such as B1, B2, B3, and B5 (with no studies identified) and B6 (with only one study available). There was also a clear predominance of studies focused on women of childbearing age and pregnant women, most of which were conducted between 2000 and 2010. In contrast, available studies on lactating women were limited to those conducted before 1990.

Given the growing burden of obesity and its potential association with micronutrient deficiencies, it is essential to investigate the prevalence and underlying determinants of B-complex vitamin deficiencies among women with obesity. Gaining a better understanding of the specific nutritional vulnerabilities in this population may inform the development of more effective and targeted public health interventions.

This study highlights a critical gap in the current literature: the lack of population-based studies with representative samples and robust sampling procedures. This limitation significantly compromises the generalizability of the findings and hinders the development of effective public health strategies. The scarcity of recent data is particularly striking in the case of lactating women, reinforcing the urgent need to include questions related to micronutrient deficiencies, especially those involving B-complex vitamins, in National Population Health Surveys in Brazil.

## Conclusion

This study systematically reviewed the prevalence of B-complex vitamin deficiencies among women of childbearing age, pregnant women, and lactating women in Brazil. Our findings reveal considerable variation in vitamin B12 deficiency rates, ranging from 0 to 29.4%, with the highest prevalence observed among women of childbearing age in a population-representative sample. In contrast, serum folate deficiency showed generally lower prevalence, although significant variations were observed depending on the cutoff points used and the populations studied.

These results emphasize the urgent need for high-quality cohort studies capable of elucidating causal pathways and long-term nutritional trends among Brazilian women of reproductive age. In addition, well-designed cross-sectional studies using robust methodologies and representative population samples are also essential to monitor current prevalence patterns and inform timely interventions. The findings reinforce the importance of developing evidence-based public health policies focused on prevention, early detection, and management of B-vitamin deficiencies. Moreover, the evidence presented here is expected to support the formulation of strategic actions aimed at preventing, treating, and reducing B-complex vitamin deficiencies in this population.

## Supplementary Information

Additional File 1: PRISMA checklist.

Additional File 2: Literature Search. Search strategy. Table 1.1 Pubmed. Table 1.2 Scopus. Table 1.3 EMBASE. Table 1.4 Web of science (main collection of WEB of SCIENCE). Table 1.5 SciELO Citation Index via Web of science (terms in English, Spanish and Portuguese language). Table 1.6 Biblioteca Digital Brasileira de teses e dissertaçoes (BDTD) – English and Portuguese language. Table 1.7 Literatura Latino Americana e do Caribe em Ciências da Saúde - LILACS (via Biblioteca Virtual em Saúde - BIREME) – English, Spanish, and Portuguese language.

Additional File 3: Global and subgroup meta-analysis of the prevalence of Vitamin B12 and Folic Acid deficiency for each population included using the I² test for heterogeneity.

## Data Availability

The datasets used and/or analyzed during the current study are available from the corresponding author upon reasonable request.

## References

[1] Black RE, Victora CG, Walker SP, Bhutta ZA, Christian P, de Onis M, et al. Maternal and child undernutrition and overweight in low-income and middle-income countries. The Lancet. 2013;382:427–51. 10.1016/S0140-6736(13)60937-X. 10.1016/S0140-6736(13)60937-X 23746772

[2] Darnton-Hill I, Mkparu U. Micronutrients in pregnancy in low- and middle-income countries. Nutrients. 2015;7:1744–68. 10.3390/nu7031744. 25763532 10.3390/nu7031744 PMC4377879

[3] WHO. Guidelines on food fortification with micronutrients. Geneva: World Health Organization; 2006.

[4] Louzada ML da C, Martins APB, Canella DS, Baraldi LG, Levy RB, Claro RM, et al. Impact of ultra-processed foods on micronutrient content in the Brazilian diet. Rev Saude Publica 2015;49:1–8. 10.1590/S0034-8910.2015049006211. 10.1590/S0034-8910.2015049006211 PMC4560336 26270019

[5] Bailey RL, West KP Jr, Black RE. The epidemiology of global micronutrient deficiencies. Ann Nutr Metab. 2015;66:22–33. 10.1159/000371618. 26045325 10.1159/000371618

[6] Darnton-Hill I. Public health aspects in the prevention and control of vitamin deficiencies. Curr Dev Nutr 2019;3:nzz075. 10.1093/cdn/nzz075. 10.1093/cdn/nzz075 PMC6775441 31598578

[7] Salam RA, Das JK, Bhutta ZA. Multiple micronutrient supplementation during pregnancy and lactation in low-to-middle-income developing country settings: impact on pregnancy outcomes. Ann Nutr Metab. 2014;65:4–12. 10.1159/000365792. 25227399 10.1159/000365792

[8] Swaminathan S, Thomas T, Kurpad AV. B-vitamin interventions for women and children in low-income populations. Curr Opin Clin Nutr Metab Care. 2015;18:295–306. 10.1097/MCO.0000000000000166. 25807352 10.1097/MCO.0000000000000166

[9] Chavarro JE, Rich-Edwards JW, Rosner BA, Willett WC. Use of multivitamins, intake of B vitamins, and risk of ovulatory infertility. Fertil Steril. 2008;89:668–76. 10.1016/j.fertnstert.2007.03.089. 17624345 10.1016/j.fertnstert.2007.03.089 PMC2366795

[10] Cueto HT, Riis AH, Hatch EE, Wise LA, Rothman KJ, Sørensen HT, et al. Folic acid supplement use and menstrual cycle characteristics: a cross-sectional study of Danish pregnancy planners. Ann Epidemiol. 2015;25:723-729.e1. 10.1016/j.annepidem.2015.05.008. 26123570 10.1016/j.annepidem.2015.05.008 PMC4567938

[11] Gaskins AJ, Chavarro JE. Diet and fertility: a review. Am J Obstet Gynecol. 2018;218:379–89. 10.1016/j.ajog.2017.08.010. 28844822 10.1016/j.ajog.2017.08.010 PMC5826784

[12] De La Calle M, Usandizaga R, Sancha M, Magdaleno F, Herranz A, Cabrillo E. Homocysteine, folic acid and B-group vitamins in obstetrics and gynaecology. European Journal of Obstetrics and Gynecology and Reproductive Biology. 2003;107:125–34. 10.1016/S0301-2115(02)00305-6. 12648856 10.1016/s0301-2115(02)00305-6

[13] Park B, Kim J. Oral contraceptive use, micronutrient deficiency, and obesity among premenopausal females in Korea: the necessity of dietary supplements and food intake improvement. PLoS ONE. 2016;11: e0158177. 10.1371/journal.pone.0158177. 27348598 10.1371/journal.pone.0158177 PMC4922824

[14] McArthur J, Tang H, Petocz P, Samman S. Biological variability and impact of oral contraceptives on vitamins b6, b12 and folate status in women of reproductive age. Nutrients. 2013;5:3634–45. 10.3390/nu5093634. 24067390 10.3390/nu5093634 PMC3798926

[15] Nunn RL, Kehoe SH, Chopra H, Sahariah SA, Gandhi M, Di Gravio C, et al. Dietary micronutrient intakes among women of reproductive age in Mumbai slums. Eur J Clin Nutr. 2019;73:1536–45. 10.1038/s41430-019-0429-6. 31148589 10.1038/s41430-019-0429-6 PMC7051904

[16] Coube M, Nikoloski Z, Mrejen M, Mossialos E. Persistent inequalities in health care services utilisation in Brazil (1998–2019). Int J Equity Health. 2023;22:1–15. 10.1186/S12939-023-01828-3/TABLES/3. 36732749 10.1186/s12939-023-01828-3 PMC9893569

[17] Landmann-Szwarcwald C, Macinko J. A panorama of health inequalities in Brazil. Int J Equity Health. 2016;15:1–3. 10.1186/S12939-016-0462-1/METRICS. 27852273 10.1186/s12939-016-0462-1 PMC5112735

[18] Canella DS, Duran AC, Claro RM. Malnutrition in all its forms and social inequalities in Brazil. Public Health Nutr. 2020;23:s29-38. 10.1017/S136898001900274X. 31591953 10.1017/S136898001900274X PMC10200539

[19] Aromataris E, Munn Z, editors. JBI Manual for Evidence Synthesis. JBI; 2020. 10.46658/JBIMES-20-01.

[20] Page MJ, McKenzie JE, Bossuyt PM, Boutron I, Hoffmann TC, Mulrow CD, et al. The PRISMA 2020 statement: an updated guideline for reporting systematic reviews. BMJ. 2021;372: n71. 10.1136/bmj.n71. 33782057 10.1136/bmj.n71 PMC8005924

[21] Mocellin MC, Curioni CC, da Silva PA, Ribas SA, Teixeira MT, de Macedo TSG, et al. Prevalence of vitamin B complex deficiencies in women in reproductive age, pregnant, or lactating woman in Brazil: a systematic review and meta-analysis protocol. Syst Rev. 2023;12:13. 10.1186/s13643-022-02136-7. 36698215 10.1186/s13643-022-02136-7 PMC9875441

[22] Ouzzani M, Hammady H, Fedorowicz Z, Elmagarmid A. Rayyan—a web and mobile app for systematic reviews. Syst Rev. 2016;5:210. 10.1186/s13643-016-0384-4. 27919275 10.1186/s13643-016-0384-4 PMC5139140

[23] Munn Z, Moola S, Lisy K, Riitano D, Tufanaru C. Methodological guidance for systematic reviews of observational epidemiological studies reporting prevalence and incidence data. Int J Evid Based Healthc. 2015;13:147–53. 26317388 10.1097/XEB.0000000000000054

[24] Daniel WW. Biostatistics - A Foundation for Analysis in the Health Sciences. 7th ed. New York: Wiley; 1999.

[25] Naing L, Winn T, Rusli BN. Practical issues in calculating the sample size for prevalence studies. Arch Orofac Sci. 2006;1:9–14.

[26] Barbosa PR, Stabler SP, Trentin R, Carvalho FR, Luchessi AD, Hirata RDC, et al. Evaluation of nutritional and genetic determinants of total homocysteine, methylmalonic acid and S-adenosylmethionine/S-adenosylhomocysteine values in Brazilian childbearing-age women. Clin Chim Acta. 2008;388:139–47. 10.1016/j.cca.2007.10.023. 18023275 10.1016/j.cca.2007.10.023

[27] Barnabé A. Prevalência Das Deficiências De Ácido Fólico, Vitamina B12 E Ferro Em Diversos Grupos Da População Brasileira, Após O Programa De Fortificação Adotado Pela Anvisa. Tese (Doutorado). Recife: Universidade Estadual de Campinas, 2014.

[28] Cavalcanti R de AS. Concentrações séricas de vitamina B2, folato intra-eritrocitário e hemoglobina em mulheres em idade fértil e sua associação com variáveis sócio-econômicas, demográficas, antropométricas e do estilo de vida. Tese (Doutorado). Recife: Universidade Federal de Pernambuco, 2018.

[29] Donangelo CM, Trugo NMF, Koury JC, Barreto Silva MI, Freitas LA, Feldheim W, et al. Iron, zinc, folate and vitamin B12 nutritional status and milk composition of low-income Brazilian mothers. Eur J Clin Nutr. 1989;43:253–66. 2661218

[30] Oliveira IO, Silva LP, Borges MC, Cruz OM, Tessmann JW, Motta JVS, et al. Interactions between lifestyle and MTHFR polymorphisms on homocysteine concentrations in young adults belonging to the 1982 Pelotas Birth Cohort. Eur J Clin Nutr. 2017;71:259–66. 10.1038/ejcn.2016.193. 27759072 10.1038/ejcn.2016.193

[31] Paiva A de A, Rondo PHC, Guerra-Shinohara EM, Silva CS. The influence of iron, vitamin B, and folate levels on soluble transferrin receptor concentration in pregnant women. Clinica Chimica Acta 2003;334:197–203. 10.1016/S0009-8981(03)00237-7. 10.1016/s0009-8981(03)00237-7 12867292

[32] Palchetti CZ, Paniz C, de Carli E, Marchioni DM, Colli C, Steluti J, et al. Association between serum unmetabolized folic acid concentrations and folic acid from fortified foods. J Am Coll Nutr. 2017;36:572–8. 10.1080/07315724.2017.1333929.Association. 28895788 10.1080/07315724.2017.1333929 PMC5697735

[33] Palchetti CZ, Steluti J, Sales CH, Fisberg RM, Marchioni DML. Folate and vitamin B12 status: temporal evaluation after mandatory fortification in Brazil. Eur J Clin Nutr. 2022;76:1266–72. 10.1038/s41430-022-01096-4. 35318452 10.1038/s41430-022-01096-4

[34] Pinto AV, Santos F, Almeida AM, Cantuaria AA. Trends of folate and vitamin B12 during pregnancy. Rev Invest Clin. 1973;25:153–8. 4722230

[35] Santos ACB dos. Frequência de consumo de frutas, hortaliças e produtos ultraprocessados e estado nutricional de gestantes de Cruzeiro do Sul, Acre. Dissertação (Mestrado). São Paulo: Universidade de São Paulo, 2016.

[36] Steluti J. Consumo dietético, variantes genéticas e relação com os níveis sanguíneos de folato, ácido fólico não metabolizado e homocisteína após a fortificação mandatória de ácido fólico: estudo de base populacional - ISA - Capital. Tese (Doutorado). São Paulo: Universidade de São Paulo, 2014.

[37] Tavares EF, Vieira-Filho JPB, Andriolo A, Perez ABA, Vergani N, Sañudo A, et al. Serum total homocysteine levels and the prevalence of folic acid deficiency and C677T mutation at the MTHFR gene in an indigenous population of Amazonia: the relationship of homocysteine with other cardiovascular risk factors. Ethn Dis. 2004;14:49–56. 15002923

[38] Tedesco AK, Biazotto R, Gebara TS e S, Cambi MPC, Baretta GAP. Pré e pós-operatório de cirurgia bariátrica: algumas alterações bioquímicas. ABCD Arq Bras Cir Dig 2016;29:67–71. 10.1590/0102-6720201600s10017. 10.1590/0102-6720201600S10017 PMC5064264 27683780

[39] Cavalcanti R de AS, Diniz A da S, de Arruda IKG. Concentrations of intra-erythrocyte folate, serum vitamin B12, and hemoglobin in women of childbearing age and associated factors. J Am Coll Nutr 2019;38:739–45. 10.1080/07315724.2019.1592725. 10.1080/07315724.2019.1592725 30990764

[40] Porto IVP. Nivéis de Homocisteína em mulheres em idade reprodutiva na cidade do Recife/PE. Tese (Doutorado). Recife: Universidade Federal de Pernambuco, 2017.

[41] Dantas JA. Consumo alimentar e concentrações intra-eritrocitárias de folato em mulheres em idade reprodutiva do Recife/PE, após a regulamentação da fortificação das farinhas de trigo e milho com ferro e ácido fólico. Dissertação (Mestrado). Recife: Universidade Federal de Pernambuco, 2009.

[42] Barnabé A, Aléssio ACM, Bittar LF, de Moraes MB, Bicudo AM, de Paula EV, et al. Folate, Vitamin B12 and homocysteine status in the post-folic acid fortification era in different subgroups of the Brazilian population attended to at a public health care center. Nutr J. 2015;14:19. 10.1186/s12937-015-0006-3. 25886278 10.1186/s12937-015-0006-3 PMC4354994

[43] Steluti J, Selhub J, Paul L, Reginaldo C, Fisberg RM, Marchioni DML. An overview of folate status in a population-based study from São Paulo, Brazil and the potential impact of 10 years of national folic acid fortification policy. Eur J Clin Nutr. 2017;71:1173–8. 10.1038/ejcn.2017.60. 28488686 10.1038/ejcn.2017.60

[44] Brito A, Mujica-Coopman MF, Olivares M, López de Romaña D, Cori H, Allen LH. Folate and vitamin B 12 status in Latin America and the Caribbean. Food Nutr Bull 2015;36:S109–18. 10.1177/0379572115585772. 10.1177/0379572115585772 26125196

[45] Sociedade Brasileira de Vegetarianismo, IBOPE. IBOPE survey shows historical growth in the number of vegetarians in the country 2018. https://www.svb.org.br/2473-vegetarians-in-brazil. Accessed 24 Sept 2021.

[46] Herrán OF, Ward JB, Villamor E. Vitamin B12 serostatus in Colombian children and adult women: results from a nationally representative survey. Public Health Nutr. 2015;18:836–43. 10.1017/S1368980014001141. 24969611 10.1017/S1368980014001141 PMC10271506

[47] Rosenthal J, Lopez-Pazos E, Dowling NF, Pfeiffer CM, Mulinare J, Vellozzi C, et al. Folate and vitamin B12 deficiency among nonpregnant women of childbearing age in Guatemala 2009–2010: prevalence and identification of vulnerable populations. Matern Child Health J. 2015;19:2272–85. 10.1007/s10995-015-1746-6.Folate. 26002178 10.1007/s10995-015-1746-6 PMC4575840

[48] Rosenthal J, Largaespada N, Bailey LB, Cannon M, Alverson CJ, Ortiz D, et al. Folate deficiency is prevalent in women of childbearing age in belize and is negatively affected by coexisting vitamin B-12 deficiency: Belize National Micronutrient Survey 2011. J Nutr. 2017;147:1183–93. 10.3945/JN.116.242628. 28404832 10.3945/jn.116.242628 PMC5548006

[49] Anaya-Loyola MA, Brito A, Villalpando S, Allen LH. Prevalence of low serum vitamin B12 in Mexican children and women: results from the first National Nutrition Survey (1999) as a basis for interventions and progress. Int J Vitam Nutr Res. 2020;90:325–32. 10.1024/0300-9831/a000579. 30987554 10.1024/0300-9831/a000579

[50] Sukumar N, Adaikalakoteswari A, Venkataraman H, Maheswaran H, Saravanan P. Vitamin B12 status in women of childbearing age in the UK and its relationship with national nutrient intake guidelines: results from two National Diet and Nutrition Surveys. BMJ Open 2016;6. 10.1136/BMJOPEN-2016-011247. 10.1136/bmjopen-2016-011247 PMC4985863 27519920

[51] Zhu JH, Hu DJ, Hao L, Zhang BL, Cogswell ME, Bailey LB, et al. Iron, folate, and B(12) deficiencies and their associations with anemia among women of childbearing age in a rural area in Northern China. Int J Vitam Nutr Res. 2010;80:144–54. 10.1024/0300-9831/A000014. 20803428 10.1024/0300-9831/a000014

[52] Abdollahi Z, Elmadfa I, Djazayeri A, Sadeghian S, Freisling H, Salehi Mazandarani F, et al. Folate, vitamin B12 and homocysteine status in women of childbearing age: baseline data of folic acid wheat flour fortification in Iran. Ann Nutr Metab. 2008;53:143–50. 10.1159/000170890. 18997463 10.1159/000170890

[53] Dang S, Yan H, Zeng L, Wang Q, Li Q, Xiao S, et al. The status of vitamin B12 and folate among Chinese women: a population-based cross-sectional study in Northwest China. PLoS ONE. 2014;9: e112586. 10.1371/journal.pone.0112586. 25390898 10.1371/journal.pone.0112586 PMC4229226

[54] Mishra J, Tomar A, Puri M, Jain A, Saraswathy KN. Trends of folate, vitamin B12, and homocysteine levels in different trimesters of pregnancy and pregnancy outcomes. Am J Hum Biol. 2020;32:1–10. 10.1002/ajhb.23388. 10.1002/ajhb.23388 31898383

[55] Finkelstein JL, Fothergill A, Krisher JT, Thomas T, Kurpad AV, Dwarkanath P. Maternal vitamin B12 deficiency and perinatal outcomes in Southern India. PLoS ONE. 2021;16: e0248145. 10.1371/journal.pone.0248145. 33822790 10.1371/journal.pone.0248145 PMC8023483

[56] Finkelstein JL, Kurpad AV, Thomas T, Srinivasan K, Duggan C. Vitamin B12 status in pregnant women and their infants in South India. Eur J Clin Nutr. 2017;71:1046–53. 10.1038/ejcn.2017.29. 28402324 10.1038/ejcn.2017.29 PMC8141370

[57] Katre P, Bhat D, Lubree H, Otiv S, Joshi S, Joglekar C, et al. Vitamin B12 and folic acid supplementation and plasma total homocysteine concentrations in pregnant Indian women with low B12 and high folate status. Asia Pac J Clin Nutr. 2010;19:335–43. 10.6133/apjcn.2010.19.3.07. 20805077

[58] Singh S, Geddam JJB, Reddy GB, Pallepogula DR, Pant HB, Neogi SB, et al. Folate, vitamin B12, ferritin and haemoglobin levels among women of childbearing age from a rural district in South India. BMC Nutr. 2017;3:50. 10.1186/s40795-017-0173-z. 32153830 10.1186/s40795-017-0173-z PMC7050838

[59] Barney A, Abraham V, Danda S, Cherian A, Vanitha S. Prevalence of vitamin B12 deficiency and its associated risk factors among pregnant women of rural South India: a community-based cross-sectional study. Indian Journal of Community Medicine. 2020;45:399–404. 10.4103/ijcm.IJCM_403_19. 33623189 10.4103/ijcm.IJCM_403_19 PMC7877432

[60] Mearns GJ, Koziol-Mclain J, Obolonkin V, Rush EC. Preventing vitamin B12 deficiency in South Asian women of childbearing age: a randomised controlled trial comparing an oral vitamin B12 supplement with B12 dietary advice. Eur J Clin Nutr. 2014;68:870–5. 10.1038/EJCN.2014.56. 24736677 10.1038/ejcn.2014.56

[61] Lamers Y. Folate recommendations for pregnancy, lactation, and infancy. Ann Nutr Metab. 2011;59:32–7. 10.1159/000332073. 22123635 10.1159/000332073

[62] WHO. Guideline: Intermittent iron and folic acid supplementation in non-anaemic pregnant women. Geneva: World Health Organization; 2013. 26110188

[63] WHO. WHO recommendations on antenatal care for a positive pregnancy experience. Geneva: World Health Organization; 2016. 28079998

[64] Brasil. Programa Nacional de Suplementação de Ferro: manual de condutas gerais. Brasília: Ministério da Saúde; 2013.

[65] BRASIL. Resolução RDC n^o^ 344, de 13 de dezembro de 2002. Aprova o Regulamento Técnico para a Fortificação das Farinhas de Trigo e das Farinhas de Milho com Ferro e Ácido Fólico. Aprova o Regulamento Técnico para a Fortificação das Farinhas de Trigo e das Farinhas de Milho com Ferro e Ácido Fólico.; 2002.

[66] Troesch B, Biesalski HK, Bos R, Buskens E, Calder PC, Saris WHM, et al. Increased intake of foods with high nutrient density can help to break the intergenerational cycle of malnutrition and obesity. Nutrients. 2015;7:6016–37. 10.3390/nu7075266. 26197337 10.3390/nu7075266 PMC4517043

[67] Agarwal S, Reider C, Brooks JR, Fulgoni VL. Comparison of Prevalence of Inadequate Nutrient Intake Based on Body Weight Status of Adults in the United States: an analysis of NHANES 2001–2008. J Am Coll Nutr. 2015;34:126–34. 10.1080/07315724.2014.901196. 25564766 10.1080/07315724.2014.901196

[68] Kaidar-Person O, Person B, Szomstein S, Rosenthal RJ. Nutritional deficiencies in morbidly obese patients: a new form of malnutrition? Part A: Vitamins Obes Surg. 2008;18:870–6. 10.1007/s11695-007-9349-y. 10.1007/s11695-007-9349-y 18465178

[69] García OP, Long KZ, Rosado JL. Impact of micronutrient deficiencies on obesity. Nutr Rev. 2009;67:559–72. 10.1111/j.1753-4887.2009.00228.x. 19785688 10.1111/j.1753-4887.2009.00228.x

[70] Capelli J de CS, Koifman S. Avaliação do estado nutricional da comunidade indígena Parkatêjê, Bom Jesus do Tocantins, Pará, Brasil. Cad Saude Publica 2001;17:433–7. 10.1590/s0102-311x2001000200018. 10.1590/s0102-311x2001000200018 11283774

[71] Ricardo CA, Ricardo FP. Sudeste do Pará. Povos indígenas no Brasil 2006–2010. São Paulo: Instituto Socioambiental; 2011. p. 435–94.

[72] Souza AI, B. Filho M, Ferreira LOC. Alterações hematológicas e gravidez. Rev Bras Hematol Hemoter 2002;24:29–36.

[73] Devalia V, Hamilton MS, Molloy AM. Guidelines for the diagnosis and treatment of cobalamin and folate disorders. Br J Haematol. 2014;166:496–513. 10.1111/bjh.12959. 24942828 10.1111/bjh.12959

[74] NIH. Folate - Health Professional Fact Sheet. National Institutes of Health, Office of Dietary Supplements 2021. https://ods.od.nih.gov/factsheets/Folate-HealthProfessional/. Accessed 30 June 2021.

[75] Green R. Indicators for assessing folate and vitamin B-12 status and for monitoring the efficacy of intervention strategies. Am J Clin Nutr. 2011;94:666S-672S. 10.3945/ajcn.110.009613. 21733877 10.3945/ajcn.110.009613 PMC3142735

[76] World Health Organization. Conclusions of a WHO technical consultation on folate and vitamin B 12 deficiencies. Food Nutr Bull. 2008;29:S238–44. 18709899 10.1177/15648265080292S129

[77] Bailey LB, Stover PJ, McNulty H, Fenech MF, Gregory JF III, Mills JL, et al. Biomarkers of nutrition for development - folate review. J Nutr. 2015;145:1636S-1680S. 10.3945/jn.114.206599. 26451605 10.3945/jn.114.206599 PMC4478945

[78] WHO. Vitamin and mineral requirements in human nutrition: report of a joint FAO/WHO expert consultation. 2nd ed. Bangkok: World Health Organization; 1998. https://doi.org/9241546123.

[79] Allen LH. Vitamin B-12. Adv Nutr. 2012;3:54–5. 10.3945/an.111.001370. 22332101 10.3945/an.111.001370 PMC3262614

[80] NIH. Vitamin B12 - fact sheet for health professionals. National Institutes of Health, Office of Dietary Supplements 2021. https://ods.od.nih.gov/factsheets/VitaminB12-HealthProfessional/#en2. Accessed 30 June 2021.

[81] Hannibal L, Lysne V, Bjørke-Monsen A-L, Behringer S, Grünert SC, Spiekerkoetter U, et al. Biomarkers and algorithms for the diagnosis of vitamin B12 deficiency. Front Mol Biosci. 2016;3:1–16. 10.3389/fmolb.2016.00027. 27446930 10.3389/fmolb.2016.00027 PMC4921487

