# Supplementary material for: Deficiency of B vitamins in women of childbearing age, pregnant, and lactating women in Brazil: a systematic review
Source: Syst Rev. 2025 May 16;14:111. doi: 10.1186/s13643-025-02861-9 (PMC12083100; doi:10.1186/s13643-025-02861-9)
Supplement: Supplementary file 2 — Additional File 2: Literature Search. Search strategy. Table 1.1 Pubmed. Table 1.2 Scopus. Table 1.3 EMBASE. Table 1.4 Web of science (main collection of WEB of SCIENCE). Table 1.5 SciELO Citation Index via Web of science (terms in English, Spanish and Portuguese language). Table 1.6 Biblioteca Digital Brasileira de teses e dissertaçoes (BDTD) – English and Portuguese language. Table 1.7 Literatura Latino Americana e do Caribe em Ciências da Saúde - LILACS (via Biblioteca Virtual em Saúde - BIREME) – English, Spanish, and Portuguese language. [file 13643_2025_2861_MOESM2_ESM.docx]

**ADDITIONAL FILE 2**

**Literature Search**

**Search strategy**

**Table 1.1 Pubmed.**

| **Deficiency of vitamin B** | Terms/MeSH  searched for in abstract and title | (((“Vitamin B Complex"[Mesh] OR “Thiamine”[Mesh] OR “Riboflavin”[Mesh] OR “Niacin*”[Mesh] OR “vitamin B6”[Mesh] OR “pantothenic acid”[Mesh] OR “biotin”[Mesh] OR “folic acid”[Mesh] OR “vitamin b12”[Mesh] OR Thiamine OR “vitamin G” OR riboflavin OR Niacin OR “nicotinic acid” OR “vitamin B*” OR pantothen* OR pyridox* OR biotin* OR “vitamin H” OR “vitamin M” OR “folic acid” OR folate OR folacin OR Cobalamin OR cyanocobalamin OR “complex* B”) **AND** (“avitaminosis”[Mesh] OR deficienc* OR Avitaminos* OR hypovitaminos* OR deficit)) **OR** ("Vitamin B Deficiency"[Mesh] OR "Vitamin B 6 Deficiency"[Mesh] OR "Folic Acid Deficiency"[Mesh] OR "Vitamin B 12 Deficiency"[Mesh]))) |
| --- | --- | --- |
|  |  | **AND** |
| **Region** | Terms/MeSH searched for in all fields | “Brazil”[Mesh] OR Brazil* OR Brasil* |
|  |  | **NOT** |
| **Exclusion** | Terms/MeSH searched for in abstract AND title | “mice”[Mesh] OR “rats”[Mesh] OR "Case-Control Studies"[Mesh] OR rats OR mouse OR mice OR “case control”  OR |
|  | Terms searched for in publication type | "Review" OR "Clinical Trial" OR "Guideline" OR "Case Reports" |

**Table 1.2 Scopus.**

| **Deficiency of vitamin B** | Terms  searched for in abstract, title and key-words | TITLE-ABS-KEY((Thiamine OR “vitamin G” OR riboflavin OR Niacin* OR “nicotinic acid” OR “vitamin B*” OR pantothen* OR pyridox* OR biotin* OR “vitamin H” OR “vitamin M” OR “folic acid” OR folate OR folacin OR Cobalamin OR cyanocobalamin OR “complex* B”) **W/5** (deficienc* OR Avitaminos* OR hypovitaminos* OR deficit)) |
| --- | --- | --- |
| **Limit** |  | Article  **AND** |
| **Region** | Terms searched for in all fields | ALL (Brazil* OR Brasil*) |
| **Limit** |  | Article |
|  |  | **AND NOT** |
| **Exclusion** | Terms searched for in abstract, title and key-words | TITLE-ABS-KEY (mice OR rats OR mouse OR “case control” OR “clinical trial” OR “case reports” OR guideline) |
| **Limit** |  | Article |

**Table 1.3 EMBASE.**

| **Deficiency of vitamin B** | Group of combination 1 | Terms searched for in abstract and title | (Thiamine OR thiamin* OR “vitamin G” OR riboflavin OR Niacin* OR “nicotinic acid” OR “vitamin B*” OR pantothen* OR “pantothenic acid” OR pyridox* OR pyridoxine OR biotin* OR biotin OR “vitamin H” OR “vitamin M” OR “folic acid” OR folate OR folacin OR Cobalamin OR cobalamin* OR cyanocobalamin OR “complex* B”):ab,ti |
| --- | --- | --- | --- |
|  |  |  | **OR** |
|  |  | Emtree terms with explode heading | (Thiamine OR riboflavin OR “nicotinic acid” OR “vitamin B group” OR cyanocobalamin OR “folic acid” OR biotin OR pyridoxine OR “pantothenic acid” OR cobalamin)/exp |
|  |  |  | **AND** |
|  | Group 2 | Terms searched for in abstract and title | (deficienc* OR deficiency OR Avitaminosis OR “vitamin deficiency” OR avitaminos* OR hypovitaminos* OR deficit):ab,ti  **OR** |
|  |  | Emtree terms with explode heading | (“folic acid deficiency” OR 'nicotinic acid deficiency' OR 'thiamine deficiency' OR 'riboflavin deficiency' OR 'vitamin B deficiency' OR 'pantothenic acid deficiency' OR 'pyridoxine deficiency' OR 'B12 deficiency')/exp |
|  |  |  | **AND** |
| **Region** | Group 3 | Terms searched in all fields | (Brazil* OR Brasil*) |
|  |  |  | **OR** |
|  |  | Emtree terms with explode heading | (Brazil OR Brazilian)/exp |
|  |  |  | **NOT** |
| **Exclusion** | Group 4 | Emtree subject headings | (Mouse OR rat OR 'case control study' OR 'clinical trial' OR 'case report' OR 'practice guideline')/exp |
|  |  |  | **OR** |
|  |  | Terms searched for in abstract and title | (mice OR rats OR mouse OR “case control” OR “clinical trial” OR “case reports” OR guideline):ab,ti |

**Table 1.4 Web of science (main collection of WEB of SCIENCE)**

| **Deficiency of vitamin B** | Terms  searched for in abstract, title and key-words | TS=((Thiamine OR “vitamin G” OR riboflavin OR Niacin* OR “nicotinic acid” OR “vitamin B*” OR pantothen* OR pyridox* OR biotin* OR “vitamin H” OR “vitamin M” OR “folic acid” OR folate OR folacin OR *Cobalamin OR “complex* B”) **NEAR/5** (deficienc* OR *vitaminos* OR deficit)) |
| --- | --- | --- |
|  |  | **AND** |
| **Region** | Terms searched for in all fields | ALL=(Brazil* OR Brasil*) |
|  |  | **NOT** |
|  |  |  |
| **Exclusion** | Terms searched for in abstract and title and key-words | TS=(mice OR rats OR mouse OR “case control” OR “clinical trial” OR “case reports” OR guideline) |

**Table 1.5 SciELO Citation Index via Web of science (terms in English, Spanish and Portuguese language)**

| **Deficiency of vitamin B** | Terms  searched for in abstract, title and key-words | TS=((Thiamine OR tiamina OR “vitamin* G” OR riboflavin* OR Niacin* OR “nicotinic acid” OR “acido nicotin*” OR “vitamin* B*” OR pantothen* OR pantoten* OR p$ridox* OR biotin* OR “vitamin* H” OR “vitamin* M” OR “folic acid” OR “acido folico” OR folat* OR folacin* OR *Cobalamin* OR “complex* B”) **AND** (deficienc* OR *vitaminos* OR deficit)) |
| --- | --- | --- |
|  |  | **AND** |
| **Region** | Terms searched for in abstract, title and key-words | TS=(Brazil* OR Brasil*) |



**Table 1.6 Biblioteca Digital Brasileira de teses e dissertaçoes (BDTD) – English and Portuguese language**

| **Deficiency of vitamin B** | Terms  searched for in all fields | Group 1 – any terms | (Todos os campos:Thiamine OR tiamina OR “vitamin* G” OR riboflavin OR riboflavina OR Niacin OR niacina OR nicotinic OR nicotínico OR “vitamina B*” OR pantothenic OR pantothenate OR pantotenico OR pantotenato OR pyridoxine OR piridoxina OR biotin OR biotina OR “vitamina H” OR “vitamina M” OR “folic acid” OR “ácido fólico” OR folato OR folate OR folacina OR folacin OR “ácido folínico” OR Cobalamin OR cobalamina OR cianocobalamina OR cyanocobalamin OR “vitamina b12” OR “vitamin B12” OR “complex* B” OR “complexo B”) |
| --- | --- | --- | --- |
|  |  |  | **AND** |
|  |  | Group 2 – any terms | (Todos os campos:deficiência OR deficiency OR avitaminosis OR avitaminose OR hipovitaminose OR hypovitaminosis OR deficit) |
|  |  |  | **NOT** |
| **Exclusion terms** |  | Group 3 – any terms | (Todos os campos:rats OR mice OR mouse OR rato OR camundongo OR “clinical trial” OR “ensaio clinico” OR “case control” OR “caso controle”) |

**Table 1.7 Literatura Latino Americana e do Caribe em Ciências da Saúde - LILACS (via Biblioteca Virtual em Saúde - BIREME) – English, Spanish and Portuguese language.**

| **Deficiency of vitamin B** | Terms  searched for in titles, abstracts and key-word | tw:(Thiamine OR tiamina OR “vitamina G” OR “vitamin G”OR riboflavin$ OR Niacin$ OR nicotinic$ OR “vitamina B” OR “vitamin B” OR pantothen$ OR pantoten$ OR pyridoxine OR piridoxina OR biotin$ OR “vitamina H” OR “vitamin M” OR “folic acid” OR “ácido fólico” OR folat$ OR folacin$ OR “ácido folínico” OR Cobalamin$ OR cianocobalamina OR cyanocobalamin OR “vitamina b12” OR “vitamin B12” OR “complex B” OR “complex B”) |
| --- | --- | --- |
|  |  | **AND** |
|  |  | tw:(deficiência OR deficiency OR avitaminos$ OR hipovitaminose OR hypovitaminosis OR deficit) |
|  |  | **AND** |
| **Region** | Terms  searched for in titles, abstracts and key-word | tw:(Brazil$ OR Brasil$) |
|  |  | **AND NOT** |
| **Exclusion terms** | Terms  searched for in titles, abstracts and key-word | tw:(rats OR mice OR mouse OR rato OR camundongo OR ratones) |
