# Supplementary material for: Deficiency of B vitamins in women of childbearing age, pregnant, and lactating women in Brazil: a systematic review
Source: Syst Rev. 2025 May 16;14:111. doi: 10.1186/s13643-025-02861-9 (PMC12083100; doi:10.1186/s13643-025-02861-9)
Supplement: Supplementary file 3 — Additional File 3: Global and subgroup meta-analysis of the prevalence of Vitamin B12 and Folic Acid deficiency for each population included using the I² test for heterogeneity. [file 13643_2025_2861_MOESM3_ESM.docx]

**ADDITIONAL FILE 3**

Global and subgroup meta-analysis of the prevalence of Vitamin B12 and Folic Acid deficiency for each population included using the I² test for heterogeneity

|  | **B12 VITAMIN** | | | **FOLIC ACID** | | |
| --- | --- | --- | --- | --- | --- | --- |
| **Subgroup** | **Number of Studies** | **Deficiency prevalence %**  **(95% CI)** | **I²** | **Number of Studies** | **Deficiency prevalence %**  **(95% CI)** | **I²** |
| **WOMEN OF CHILDBEARING AGE** | | | | | | |
| **Region** | | | | | | |
| Southeast | 4 | 12.0 (3.0-26.0) | 93.0 | 4 | <CP: 1.0 (0.0-3.0)  >CP: 6.0 (0.0-25.0) | 0  96.6 |
| North East | 1 | 7.0 (6.0-9.0) | - | 0 | **-** | **-** |
| Midwest | 1 | 0.0 (0.0-12.0) | - | 1 | <CP: 10.0 (2.0-27.0)  >CP: 10.0 (2.0-27.0) | **-** |
| North | 1 | 0.0 (0.0-13.0) | - | 1 | <CP: 15.0 (4.0-35.0)  >CP: 15.0 (4.0-35.0) | **-** |
| South | - | - | - | 1 | <CP: 0.0 (0.0-1.0)  >CP: 3.0 (2.0-4.0) | **-** |
| **Age** | | | | | | |
| ≥19 years | 4 | 6.0 (0.0-22.0) | 93.0 | 5 | <CP: 2.0 (0.0-5.0)  >CP: 8.0 (1.0-22.0) | 77.6  95.8 |
| 10-49 years | 2 | 9.0 (7.0-10.0) | 0.0 | 1 | <CP: 1.0 (0.0-4.0)  >CP: 1.0 (0.0-4.0) | - |
| Not specified | 1 | 0.0 (0.0-12.0) | - | 1 | <CP: 10.0 (2.0-27.0)  >CP: 10.0 (2.0-27.0) | - |
| **Data collection year** | | | | | | |
| < 1990 | 1 | 0.0 (0.0-12.0) | - | 1 | <CP: 10.0 (2.0-27.0)  >CP: 10.0 (2.0-27.0) | - |
| 2000-2010 | 4 | 9.0 (3.0-17.0) | 90.5 | 4 | <CP: 2.0 (0.0-5.0)  >CP: 11.0 (1.0-27.0) | 83.2  0.0 |
| 2010-2020 | 2 | 12.0 (7.0-18.0) | 0.0 | 2 | <CP: 1.0 (0.0-4.0)  >CP: 1.0 (0.0-4.0) | 0.0  0.0 |
| **Mandatory fortification of flour with folic acid** | | | | | | |
| < 2004 | - | - | - | 2 | <CP: 3.0 (0.0-7.0)  >CP: 30.0 (22.0-38.0) | 0.0  0.0 |
| ≥ 2004 | - | - | - | 5 | <CP: 1.0 (0.0-4.0)  >CP: 2.0 (1.0-5.0) | 77.2  58.7 |
| **Deficiency cutoff point** | | | | | | |
| ≤ 140 pg/mL | 1 | 0.0 (0.0-12.0) | - | - | - |  |
| ≤ 190 pg/mL | 1 | 0.0 (0.0-13.0) | - | - | - |  |
| ≤ 200 pg/mL | 5 | 11.0 (4.0-20.0) | 94.0 | - | - |  |
| < 3 ng/mL | - | - |  | 5 | 2.0 (0.0-5.0) | 79.0 |
| < 4 ng/mL | - | - |  | 2 | 2.0 (0.0-6.0) | 0.0 |
| **OVERALL ANALYSIS** | 7 | 7.0 (2.0-14.0) | **92.1** | 7 | <CP: 2.0 (0.0-5.0)  >CP: 7.0 (2.0-15.0) | 78.0  94.1 |
| **PREGNANT WOMEN** | | | | | | |
| **Region** | | | | | | |
| Southeast | 2 | 9.0 (6.0-12.0) | 0.0 | - | - | - |
| Midwest | 1 | 4.0 (0.0-9.0) | - | - | - | - |
| **Data collection year** | | | | | | |
| < 1990 | 1 | 4.0 (0.0-9.0) | - | - | - | - |
| 2000-2010 | 2 | 9.0 (6.0-12.0) | 0.0 | - | - | - |
| **Deficiency cutoff point** | | | | | | |
| ≤ 140 pg/mL | 1 | 4.0 (0.0-9.0) | - | - | - | - |
| ≤ 200 pg/mL | 2 | 9.0 (6.0-12.0) | 0.0 | - | - | - |
| < 3 ng/mL | - | - | - | 1 | 0.0 (0.0-1.0) | - |
| < 4 ng/mL | - | - | - | 2 | 2.0 (0.0-3.0) | 0.0 |
| **OVERALL ANALYSIS** | 3 | 8.0 (2.0-16.0) | 75.7 | 3 | 5.0 (0.0-14.0) | 93.3 |
| **LACTATING WOMEN** | | | | | | |
| **Region** | | | | | | |
| Southeast | 2 | 6.0 (2.0-11.0) | 0.0 | 2 | 6.0 (2.0-12.0) | 0.0 |
| Midwest | 1 | 6.0 (1.0-19.0) | - | 1 | 54.0 (37.0-71.0) | - |
| **Data collection year** | | | | | | |
| < 1990 | 2 | 9.0 (4.0-15.0) | 0.0 | 2 | 29.0 (20.0-38.0) | 0.0 |
| 2000-2010 | 1 | 2.0 (0.0-10.0) | - | 1 | 0.0 (0.0-7.0) | - |
| **Deficiency cutoff point** | | | | | | |
| < 3 ng/mL | - | - | - | 1 | 17.0 (9.0-28.0) | - |
| < 4 ng/mL | - | - | - | 2 | 12.0 (6.0-20.0) | 0.0 |
| **OVERALL ANALYSIS** | 3 | 6.0 (0.0-20.0) | 84.4 | 3 | 18.0 (0.0-49.0) | 94.3 |
| **WOMEN OF CHILDBEARING AGE WITH OBESITY** | | | | | | |
| **Deficiency cutoff point** | | | | | | |
| < 3 ng/mL | - | - | - | 2 | 1.0 (0.0-3.0) | 0.0 |
| < 4 ng/mL | - | - | - | 2 | 7.0 (3.0-12.0) | 0.0 |

Legend: 95%CI: Confidence interval; I²: Inconsistency; <CP: lowest cutoff points used (3 and 4 ng/mL); >CP: highest cutoff points used (3, 4 and 6 ng/mL).

Source: from the author.
